# Exosome‐delivered circRNA promotes glycolysis to induce chemoresistance through the miR‐122‐PKM2 axis in colorectal cancer

**DOI:** 10.1002/1878-0261.12629

**Published:** 2020-01-24

**Authors:** Xinyi Wang, Haiyang Zhang, Haiou Yang, Ming Bai, Tao Ning, Ting Deng, Rui Liu, Qian Fan, Kegan Zhu, Jialu Li, Yang Zhan, Guoguang Ying, Yi Ba

**Affiliations:** ^1^ Tianjin Medical University Cancer Institute and Hospital National Clinical Research Center for Cancer Tianjin's Clinical Research Center for Cancer Key Laboratory of Cancer Prevention and Therapy Tianjin China; ^2^ Division of Gastroenterology and Hepatology Shanghai Institute of Digestive Disease China; ^3^ Key Laboratory of Gastroenterology and Hepatology Ministry of Health Shanghai Jiao‐Tong University School of Medicine Renji Hospital China

**Keywords:** aerobic glycolysis, chemoresistance, circRNA, exosome, hsa_circ_0005963, PKM2

## Abstract

Malignant tumors, including colorectal cancer (CRC), usually rely on ATP generation through aerobic glycolysis for both rapid growth and chemotherapy resistance. The M2 isoform of pyruvate kinase (PKM2) has a key role in catalyzing glycolysis, and PKM2 expression varies even within a single tumor. In this study, we confirmed that expression of PKM2 is heterogeneous in CRC cells, namely high in oxaliplatin‐resistant cells but relatively low in sensitive cells, and found that chemoresistant cells had enhanced glycolysis and ATP production. In addition, we report a PKM2‐dependent mechanism through which chemosensitive cells may gradually transform into chemoresistant cells. The circular RNA hsa_circ_0005963 (termed ciRS‐122 in this study), which was determined to be a sponge for the PKM2‐targeting miR‐122, was positively correlated with chemoresistance. *In vitro* and *in vivo* studies showed that exosomes from oxaliplatin‐resistant cells delivered ciRS‐122 to sensitive cells, thereby promoting glycolysis and drug resistance through miR‐122 sponging and PKM2 upregulation. Moreover, si‐ciRS‐122 transported by exosomes could suppress glycolysis and reverse resistance to oxaliplatin by regulating the ciRS‐122–miR‐122–PKM2 pathway *in vivo*. Exosomes derived from chemoresistant CRC cells could transfer ciRS‐122 across cells and promote glycolysis to reduce drug susceptibility in chemosensitive cells. This intercellular signal delivery suggests a potential novel therapeutic target and establishes a foundation for future clinical applications in drug‐resistant CRC.

## Graphical abstract

Exosomes from oxaliplatin‐resistant colorectal cancer (CRC) cells transferred ciRS‐122 to oxaliplatin‐sensitive cells, enhancing glycolysis and drug resistance by promoting PKM2 expression. Furthermore, ciRS‐122 targeting through exosome‐delivered small interfering (si)RNA *in vivo* enhanced the drug response, indicating a novel potential approach for the reversion of oxaliplatin resistance in CRC.

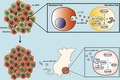

**Abbreviations**
A: absorbance
ABC: ATP‐binding cassette
CG : control group
circRNA: circular RNA
ciRS‐122: hsa_circ_0005963 was a sponge for miR‐122 and named ciRS‐122 in the study
CRC: colorectal cancer
*C*_t_: cycle threshold
EG : experimental group
IHC: immunohistochemistry
lncRNA: long non‐coding RNA
L‐OHP : oxaliplatin
miRNA : microRNA
NC : negative control
NTA: nanoparticle tracking analysis
PI : propidium iodide
PKM2: M2 isoform of pyruvate kinase
RT: room temperature
siRNA : short interfering RNA
TEM: transmission electron microscopy
WB: western blot

## 1 Introduction

Malignant solid tumors take up large amounts of glucose to produce lactate even in the presence of oxygen compared with what is observed in the surrounding tissue; this phenomenon has been termed ‘aerobic glycolysis’ (Liberti and Locasale, 2016). A key control for metabolizing glucose by either glycolysis or the oxidative phosphorylation process is regulation of the flux of glycolysis. Pyruvate kinase catalyzes the final reaction in glycolysis by transferring the high‐energy phosphate from phosphoenolpyruvate to ADP to produce ATP and pyruvate. Among the four isoforms of pyruvate kinase, the M2 isoform (PKM2) is the dominant type in proliferating and cancer cells (Wong et al., 2015). Known as heterogeneity, clonal variation and microenvironmental influence on cancer cells result in distinct populations of cells, even within a single tumor (Prasetyanti and Medema, 2017). The glycolytic phenotypes do not display homogeneously in tumors because of the diverse expression of PKM2. When PKM2 is overexpressed, the glycolytic rate is high, and most glucose is converted to lactate with a rapid production of ATP (Chaneton and Gottlieb, 2012).

Multiple biological activities within cells require the consumption of ATP. In cancer cells, some ATP‐binding cassette (ABC) drug efflux pumps embedded across the membranes play a pivotal role in survival (Li *et al.*, 2016). With increasing intracellular ATP, the transporters could obtain more energy to pump the drugs out of the cells to prevent intracellular drugs from accumulating and damaging the cells. In an individual or even a single tumor, the capability of ATP generation varies among distinct sets of cells due to the diversity of glycolysis rates; hence, the ability to reduce drugs in the cytoplasm could be different (Zhou *et al.*, 2012). In other words, drug‐sensitive cells and drug‐resistant cells might exist simultaneously within the same tumor. Nevertheless, the development of the ABC transporter as a therapeutic target has been unsuccessful in 30 years of research (Robey *et al.*, 2018). Chemoresistance remains a continuing challenge in the field of cancer therapy.

It remains unclear how drug‐sensitive cells become drug‐resistant ones as a tumor progresses in an individual. Exosomes have been noted as carriers for intercellular signal transduction in recent years (Zhang *et al.*, 2017). Exosomes are extracellular vesicles secreted by various cells, with diameters ranging from 30 to 100 nm, delivering micro (mi)RNA, long non‐coding (lnc)RNA, circular (circ)RNA and proteins (Mathieu *et al.*, 2019; Xu *et al.*, 2018). It was demonstrated that some horizontal interactions via exosomes modulated drug response in a heterogeneous tumor *in vivo*. Drug‐resistant and ‐sensitive clones coexisted initially, but most cells eventually became poorly responsive to chemotherapy (Sharma, 2017). Among the various cargos carried in exosomes, circRNA are a novel class of noncoding RNA that are exceptionally stable. Some circRNA have been shown to function as efficient micro (mi)RNA sponges with gene‐regulatory potential involved in cancer (Kristensen *et al.*, 2018). Research examining circRNA is steadily increasing and is predominantly about intracellular signal transduction but largely has not investigated intercellular regulation (Arnaiz *et al.*, 2018).

The current study is designed to research the mechanism of drug resistance in colorectal cancer (CRC). The expression pattern of PKM2 was heterogeneous even within a single tumor, being high in drug‐resistant cells but relatively low in drug‐sensitive cells. Glycolysis was also stronger in drug‐resistant cells, with more ATP production being observed. A panel of circRNA was found to be dysregulated; among these circRNA, ciRS‐122 was predicted to act as a sponge of miR‐122 in oxaliplatin‐resistant CRC cells. Subsequently, it was verified that the expression level of ciRS‐122 in serum exosomes was positively correlated with chemoresistance. An *in vitro* and *in vivo* study demonstrated that exosomes from drug‐resistant cells could deliver ciRS‐122 to drug‐sensitive cells, in which glycolysis and drug resistance were enhanced by decreasing miR‐122 and upregulating PKM2. In addition, the inhibition of ciRS‐122 suppressed glycolysis and reversed the resistance to oxaliplatin in CRC. The results of this study indicate that exosomes play a key role in mediating chemoresistance from drug‐resistant cells to drug‐sensitive cells by delivering circRNA, and circRNA serve as a potentially novel target for the treatment of drug‐resistant CRC.

## 2 Materials and methods

### 2.1 Human tissue and immunohistochemistry

All human CRC tissue samples were obtained from Tianjin Medical University Cancer Institute and Hospital. The tumors were fixed in 4% paraformaldehyde, embedded in paraffin, sectioned, and then stained with anti‐PKM2 antibodies (Santa Cruz, CA, USA). The experiments were undertaken with the understanding and written consent of each subject. The study methodologies conformed to the standards set by the Declaration of Helsinki, and the Ethics Committee of Tianjin Medical University Cancer Institute and Hospital approved all aspects of this study.

### 2.2 Animals

Female nude mice (BALB/c‐nu, 4 weeks) purchased from GemPharmatech Co., Ltd (Jiangsu, China) were fed in a special pathogen‐free animal facility and allowed to eat and drink *ad libitum*. All the experimental procedures were performed in line with protocols approved by the Institutional Animal Care and Research Advisory Committee of Tianjin Medical University Cancer Institute and Hospital.

### 2.3 Cell culture

The human CRC cell line SW480 and HCT116, and the human embryo kidney epithelial cell line HEK293T were acquired from the cell bank of the Chinese Academy of Sciences (Shanghai, China). The human oxaliplatin‐resistant CRC cell line SW480/oxaliplatin (L‐OHP) and HCT116/L‐OHP were established via gradual exposure of the parent cells to increasing concentrations of oxaliplatin in regular cell culture conditions for the selection of resistant cells. HEK293T was cultured in DMEM (Gibco, Grand Island, NY, USA) and the others were in 1640 (Gibco). All the cells were cultured with 10% FBS (Gibco) and 1% penicillin/streptomycin (Solarbio, Beijing, China) in a humidified incubator at 37 °C with 5% CO_2_.

### 2.4 Cell transfection

Different cells were seeded in different plates and transfected with Lipofectamine 2000 (Invitrogen, Carlsbad, CA, USA) and Opti‐MEM (Gibco) in accordance with the manufacturer's instructions. Mimics or inhibitors of miR‐122 (RiboBio, Guangzhou, China) were used for miR‐122 upregulation or downregulation. Short interfering RNA of ciRS‐122 (RiboBio; target sequence: CGAAGAAACCTCCACAGCT) or PKM2 (Santa Cruz; sc‐62820) were applied to knock down ciRS‐122 or PKM2. Circular RS‐122 or PKM2 OE plasmids (GeneChem, Shanghai, China) were utilized for overexpression of ciRS‐122 or PKM2. Cells were washed with 1× PBS, and the medium was replaced by complete medium 4–6 h after transfection.

### 2.5 RNA sequence analysis

HCT116 and HCT116/L‐OHP cells were utilized for RNA sequencing. The assays were conducted on the Illumina sequencing platform by Genedenovo Biotechnology Co., Ltd. (Guangzhou, China). To identify differentially expressed circRNA between samples, the edgeR package (http://www.r-project.org/) was applied. Comparing HCT116/L‐OHP with HCT116 cells, significantly differentially expressed circRNA were screened through fold change ≥ 2 and a *P*‐value < 0.05.

### 2.6 Isolation of exosomes from cell culture medium and serum

Medium with 10% exosome‐free FBS was prepared via 100 000 ***g*** ultracentrifugation (Rotor: SW 32 Ti, Beckman Coulter, Brea, CA, USA) of complete medium for 18 h. After incubation in the conditioned medium for 24–48 h, the medium was centrifuged at 300 ***g*** and 3000 ***g*** to discard cell debris. The supernatant was then centrifuged at 10 000 ***g*** for 30 min to remove large‐sized shedding vesicles. Eventually, the supernatant was ultracentrifuged at 110 000 ***g*** for 70 min (Rotor: Beckman Coulter SW 41 Ti), and exosomes were contained in the pellet, which was resuspended in 1× PBS (Ramirez *et al.*, 2018) and filtered with 0.2‐μm filters. Generally, 100–200 μg of exosomes could be extracted from a 10‐cm dish of cell culture medium. All steps were performed at 4 °C. Serum exosomes were isolated by applying the Total Exosome Isolation Kit (Invitrogen).

### 2.7 Transmission electron microscopy (TEM)

Through a series of special processing (detailed in our previous study, Wang *et al.*, 2018), exosomes were observed and photographed with an FEI Tecnai T20 transmission electron microscope (Thermo, Waltham, MA, USA).

### 2.8 Nanoparticle tracking analysis (NTA)

Sizes and numbers of exosomes were tracked via the NanoSight NS300 system (NanoSight Technology, Malvern, UK). Exosomes were resuspended in PBS at a concentration of 5 μg·mL^−1^ and further diluted 100‐ to 500‐fold to achieve between 20 and 100 objects per frame. Subsequently, samples were injected into sample chambers at room temperature (RT), each of which was configured with a 488‐nm laser and a high‐sensitivity sCMOS camera. At least 200 completed tracks were analyzed per video. All the data were analyzed by the nta analytical software (version 2.3; Thery and Witwer, 2018).

### 2.9 Proteinase and RNase protection assay

Isolated exosomes were pretreated with or without 0.1% Triton X‐100 (Sigma, Santa Clara, CA, USA) and incubated with RNase A (0.5 μg·μL^−1^; Solarbio) at 37 °C for 20 min or incubated with proteinase K (0.05 μg·μL^−1^; Solarbio) at 37 °C for 10 min first, due to some RNA exterior to exosomes that were protected by protein complexes. Proteinase K activity was then inactivated via mixing with 5 mm PMSF at RT for 10 min and 90 °C for 5 min, followed by RNase A addition. Ultimately, RNA were extracted from the exosomes and detected by RT‐qPCR (Mateescu and Kowal, 2017).

### 2.10 PKH26 staining

The PKH26 Red Fluorescent Cell Linker Kit (Sigma) was utilized for exosome staining. Fifty microgram of exosomes (quantified by mass concentration with NanoDrop 2000 Spectrophotometer, Thermo, Waltham, MA, USA) resuspended in 100 μL of diluent C was mixed with 100 μL PKH26 dye solution (4 × 10^−6^ m) and incubated for 1–5 min, which was stopped by adding 200 μL of serum. The labeled exosomes were then washed twice with PBS and coincubated with recipient cells in one well of a 6‐well plate for 2–24 h before imaging was performed.

### 2.11 Protein extraction and western blotting

Proteins were isolated with SDS lysis buffer (50 mm Tris‐HCl pH 6.8, 2.2% SDS, 5.5% glycerol and 1 mm PMSF) from cultured cells, which were washed twice with 1× PBS first. Exosomes were washed with 1× PBS and resuspended in SDS lysis buffer. Small pieces of tissues were ground with liquid nitrogen and mixed with SDS lysis buffer followed by centrifugation at 16 000 ***g*** for 30 min and supernatant collection. All steps were performed at 4 °C. Subsequently, the lysates were heated at 95 °C for 10 min, quantified by NanoDrop 2000, loaded with 16× β‐Blue (20% β‐mercaptoethanol and 0.08% bromophenol blue) and stored at −80 °C. Fifty micrograms of proteins extracted from cultured cells, exosomes or tissues were loaded in each well, separated via SDS‐PAGE and transferred onto polyvinylidene fluoride membranes (Roche, Basel, Switzerland). Antibodies including anti‐CD63 (1 : 200; Santa Cruz, sc‐5275), anti‐TSG101 (1 : 200; Santa Cruz, sc‐7964), anti‐albumin (Abcam, Cambridge, UK; ab207327), anti‐calnexin (Abcam; ab213243) and anti‐PKM2 (1 : 500; Santa Cruz, sc‐365684) were applied to analyze different proteins, and β‐actin antibody (1 : 500; Santa Cruz, sc‐47778) was utilized for normalization.

### 2.12 RNA isolation and RT‐qPCR

Total RNA was extracted with TRIzol reagent (Invitrogen) from cultured cells (washed twice with 1× PBS first), exosomes and tissues. A total of 1000 ng of RNA from cultured cells or tissues or 500 ng from exosomes were utilized for reverse transcription PCR (Eppendorf AG 22331 Hamburg, Germany) to synthesize cDNA via applying avian myeloblastosis virus reverse transcriptase (TaKaRa, Osaka, Japan). Afterwards, 1 μL of cDNA was used for real‐time qPCR (Bio‐Rad CFX96, Hercules, CA, USA). Quantification of miR‐122 was performed using TaqMan miRNA probes (Applied Biosystems, Foster city, CA, USA; 4427975, 002245) and normalized to the internal control U6 small nuclear RNA (Applied Biosystems, 4427975, 001973), and circRNA and mRNA levels were normalized to β‐actin. The relative levels of genes were calculated with the equation 2 ${}^{-\Delta C_{t}}$, in which $\Delta C_{t}\left(\text{cycle}\;\text{threshold}\right)=C_{t_{\text{gene}}}-C_{t_{\text{control}}}$. All genes were assayed at least in triplicate. Primers of ciRS‐122, PKM2 and β‐actin were as follows:

ciRS‐122 primers

Forward 5′‐TACCCAGTTTATGGGGGTTGT‐3′

Reverse 5′‐AAACTGACAAGCAACAAGGCAC‐3′

PKM2 primers

Forward 5′‐TGACGAGAACATCCTGTGGC‐3′

Reverse 5′‐AGCACAGATGACAGGCTTCC‐3′

β‐Actin primers

Forward 5′‐GGCTGTGCTATCCCTGTACG‐3′

Reverse 5′‐CTTGATCTTCATTGTGCTGGGTG‐3′

### 2.13 Biotinylated miRNA capture and RNA pulldown

Biotin‐labeled miR‐122 (GenePharma, Shanghai, China) was transfected into HEK293T cells; 24 h later, cells were lysed by lysis buffer containing 20 mm Tris‐HCl pH 7.5, 150 mm NaCl, 1 mm EDTA, 0.5% NP‐40, 0.5 mm DTT and 100 U·mL^−1^ SUPERASin. Dynabeads™ magnetic beads (Invitrogen) were prepared in advance following the instructions. Cell lysate was centrifuged, and the supernatant was mixed with the magnetic beads and incubated with gentle rotation for 15–30 min at RT. The biotin‐coupled RNA‐coated beads were then washed 3–4 times and resuspended in TRIzol reagent for RNA identification.

### 2.14 Bioinformatics analysis

Target prediction of miRNA was performed with the algorithms from targetscanhuman 7.2 (http://www.targetscan.org/vert_72/). Interactions between miRNA and circRNA were analyzed via starbase v3.0 (http://starbase.sysu.edu.cn/) and rnahybrid (https://bibiserv.cebitec.uni-bielefeld.de/rnahybrid).

### 2.15 Luciferase reporter assay

The reporter plasmids p‐MIR‐ciRS‐122 containing miR‐122 binding regions 1 or 2 were designed by GenScript (Nanjing, China). Parts of the wild‐type and mutant sequences of miR‐122 binding regions 1 or 2 were cloned into the firefly luciferase reporter. Details were described in our previous research (Zhang *et al.*, 2019a).

### 2.16 CCK‐8 cell viability assay

Cells were cultured in 96‐well plates followed by different pretreatments and exposure to L‐OHP (Hengrui Pharma, Jiangsu, China) at various doses for 48 h. Cell viability was detected with CCK‐8 (Solarbio). The absorbance (A) value was examined at a wavelength of 450 nm on a microplate reader (Thermo). Assays were conducted in triplicate at least. The following equation was applied for calculation:

$$\text{Inhibition}\;\text{ratio}=\left(A_{\text{control}}-A_{\text{experiment}}\right)/\left(A_{\text{control}}-A_{\text{blank}}\right)\times 100\%.$$

### 2.17 Flow cytometry

SW480 and SW480/L‐OHP cells were preprocessed and incubated with various concentrations (30 μg·mL^−1^ for SW480 and 60 μg·mL^−1^ for SW480/L‐OHP) of oxaliplatin for 48 h. Subsequently, both the attached and floating cells were collected and labeled with Annexin V‐FITC and PI (propidium iodide; BD Biosciences, Franklin Lakes, NJ, USA). Apoptotic cells were quantified by a fluorescence‐activated cell sorting flow cytometer (BD Biosciences).

### 2.18 ATP measurement and ATP loading

Cells were implanted in opaque‐walled 96‐well plates (Corning, Toledo, OH, USA), and control wells containing medium without cells were also prepared to obtain a value for background luminescence. A CellTiter‐Glo® Luminescent Cell Viability Assay (Promega, Madison, WI, USA) was applied for ATP measurement. After pretreatment, the plate and its contents were equilibrated at RT for approximately 30 min. Then, 100 μL of CellTiter‐Glo® reagent was added to each well, which was mixed for 2 min on an orbital shaker to induce cell lysis and incubated at RT for 10 min to stabilize the luminescent signal before the luminescence record via a microplate reader (BioTek Synergy H1, Winooski, VT, USA). The average ATP content per cell was calculated in accordance with the cell number in each well.

Encapsulation of ATP with liposomes was carried out according to a published protocol (Verma *et al.*, 2005). Lipofectamine 2000 (Invitrogen) was employed to deliver ATP (Solarbio) into cells as described above.

### 2.19 Glucose uptake and lactate product assay

The relative glucose uptake was assessed by measuring the glucose concentration in medium (One Touch, Johnson & Johnson, New Brunswick, NJ, USA; Zhang *et al.*, 2019b), and the lactate production in the medium was detected using a Lactate Assay Kit according to the manufacturer's protocol (Solarbio, Beijing, China). All metabolic indicators were calibrated with the total number of cells before these tests were performed.

### 2.20 Establishment of tumors in nude mice

A total of 1 × 10^7^ SW480 or SW480/L‐OHP cells were injected into several mice subcutaneously. Tumors were removed after a month and divided into small pieces 2 mm in diameter, which were then implanted into the hypodermic areas in the right or left inguinal regions of the mice in every group.

### 2.21 Statistical analyses

All the data in our study are based on three independent experiments at least and exhibited as the mean ± SEM. *P* < 0.05 was regarded as statistically significant via Student's *t*‐test: *P < 0.05; **P < 0.01; and ***P < 0.001.

## 3 Results

### 3.1 Expression patterns of PKM2 and ciRS‐122 in CRC

**Figure 1:**
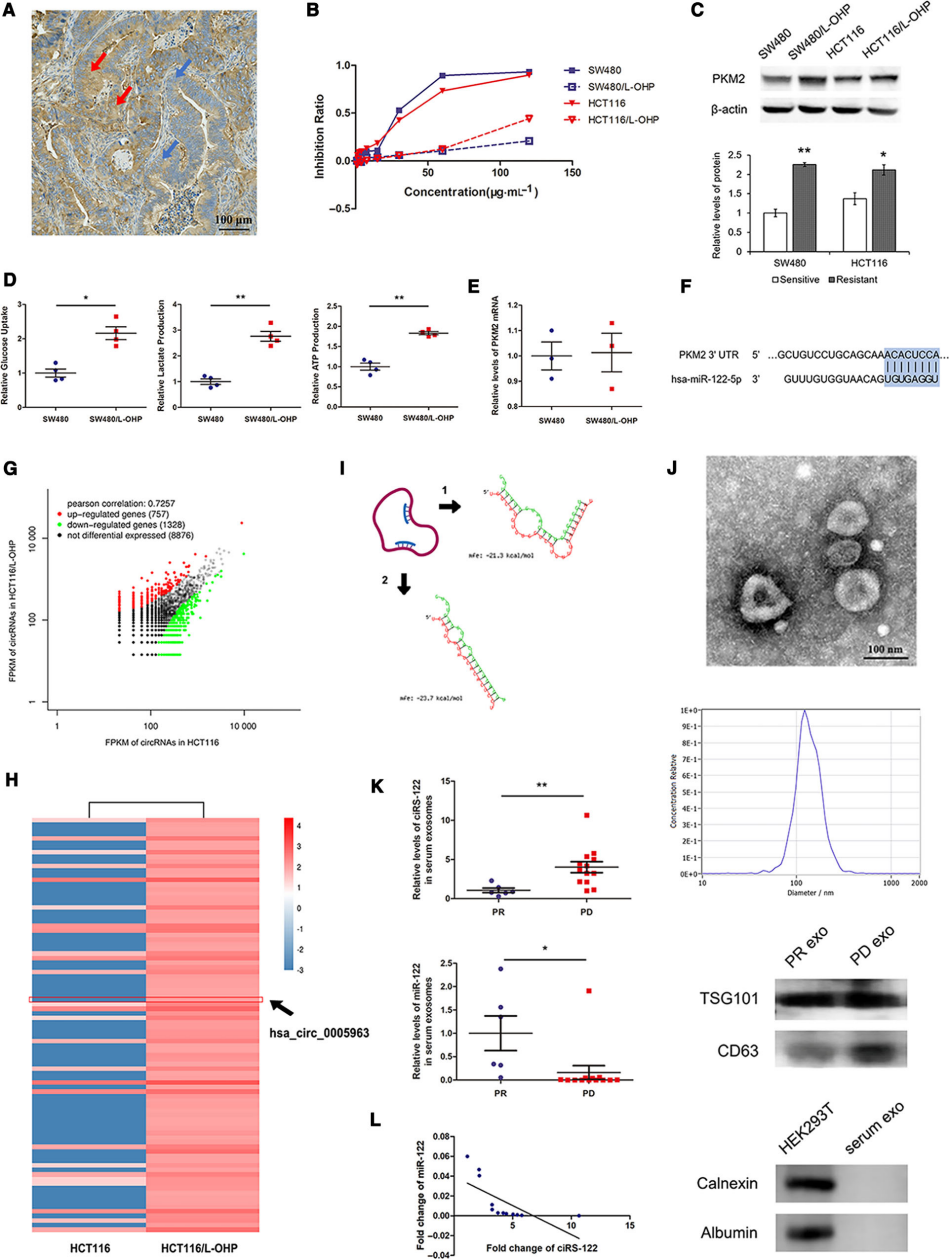
Expression patterns of PKM2 and ciRS‐122 in CRC. (A) The heterogeneous expression of PKM2 in CRC tissues by IHC, red arrows for high expression, blue arrows for low expression. (B) Validation of oxaliplatin resistance of cell lines by CCK‐8 assay. (C) Western blot (WB) analysis of PKM2 expression in cell lines (*n* = 3, mean ± SEM, *t*‐test). (D) The relative glucose uptake, lactate production and ATP production in cells (*n* = 4, mean ± SEM, *t*‐test). (E) RT‐qPCR quantification of PKM2 mRNA in cells (*n* = 3, mean ± SEM, *t*‐test). (F) Bioinformatic prediction of the binding site of miR‐122 in the PKM2 3′ UTR. (G) Preliminary screening of oxaliplatin‐resistance‐related circRNAs in CRC cells by RNA sequence. (H) Heatmap of the remarkably upregulated circRNAs in Fig. 1G. (I) Bioinformatic analysis of two putative binding regions between ciRS‐122 and miR‐122. (J) TEM, NTA and WB confirmation of human serum exosomes. (K) RT‐qPCR quantification of the expression levels of ciRS‐122 and miR‐122 within serum exosomes in PR (*n* = 6) and PD (*n* = 13) groups (mean ± SEM, *t*‐test). (L) Correlation analysis between ciRS‐122 and miR‐122 in serum exosomes. *P < 0.05; **P < 0.01.

To confirm the heterogeneity of PKM2 in tumors, seven human CRC tumor tissues were utilized for immunohistochemistry (IHC). The expression of PKM2 varied (Fig. S1) even within a single tumor (Fig. 1A). Afterwards, two types of oxaliplatin‐resistant cell lines were verified by the CCK‐8 assay (Fig. 1B); PKM2 was highly expressed in drug‐resistant cells but was relatively low in drug‐sensitive cells (Fig. 1C). The relative glucose uptake and lactate production were also stronger in oxaliplatin‐resistant cells with more ATP production (Fig. 1D), representative of the more active glycolysis. Subsequently, the levels of PKM2 mRNA were detected to explore why the expression of PKM2 was in chemoresistant and chemosensitive cells. Nevertheless, there were no significant differences between mRNAs (Fig. 1E), which indicated a potential posttranscriptional regulation. By bioinformatics analysis, it was found that the 3′ UTR of human PKM2 harbored a putative binding site of miR‐122 (Fig. 1F). A panel of circRNA was determined to be dysregulated in the oxaliplatin‐resistant CRC cells (Fig. 1G). Among the remarkably upregulated circRNA (Fig. 1H), hsa_circ_0005963 was predicted to interact with miR‐122 via two potential binding regions (Fig. 1I) and is named ciRS‐122 in this study. In other words, ciRS‐122 might enhance the expression of PKM2 by sponging miR‐122 in CRC at the posttranscriptional level. For further investigation of the clinical correlation between ciRS‐122 and miR‐122, exosomes were isolated from the serum of oxaliplatin‐sensitive patients (PR, *n* = 6) and oxaliplatin‐resistant patients (PD, *n* = 13) and observed to have a typical round morphology, 50–150 nm in diameter. Exosomes were enriched for CD63 and TSG101 while not exhibiting albumin or calnexin (Fig. 1J). The expression level of ciRS‐122 within exosomes was higher in the PD group, whereas miR‐122 was notably suppressed compared with the PR group (Fig. 1K), revealing a negative relationship between ciRS‐122 and miR‐122 in serum exosomes (Fig. 1L). These data suggest that the expression of PKM2 is closely related to oxaliplatin resistance in CRC and may be positively regulated by exosome‐delivered ciRS‐122.

### 3.2 Effects of chemoresistant cell‐derived exosomes on sensitive cells

**Figure 2:**
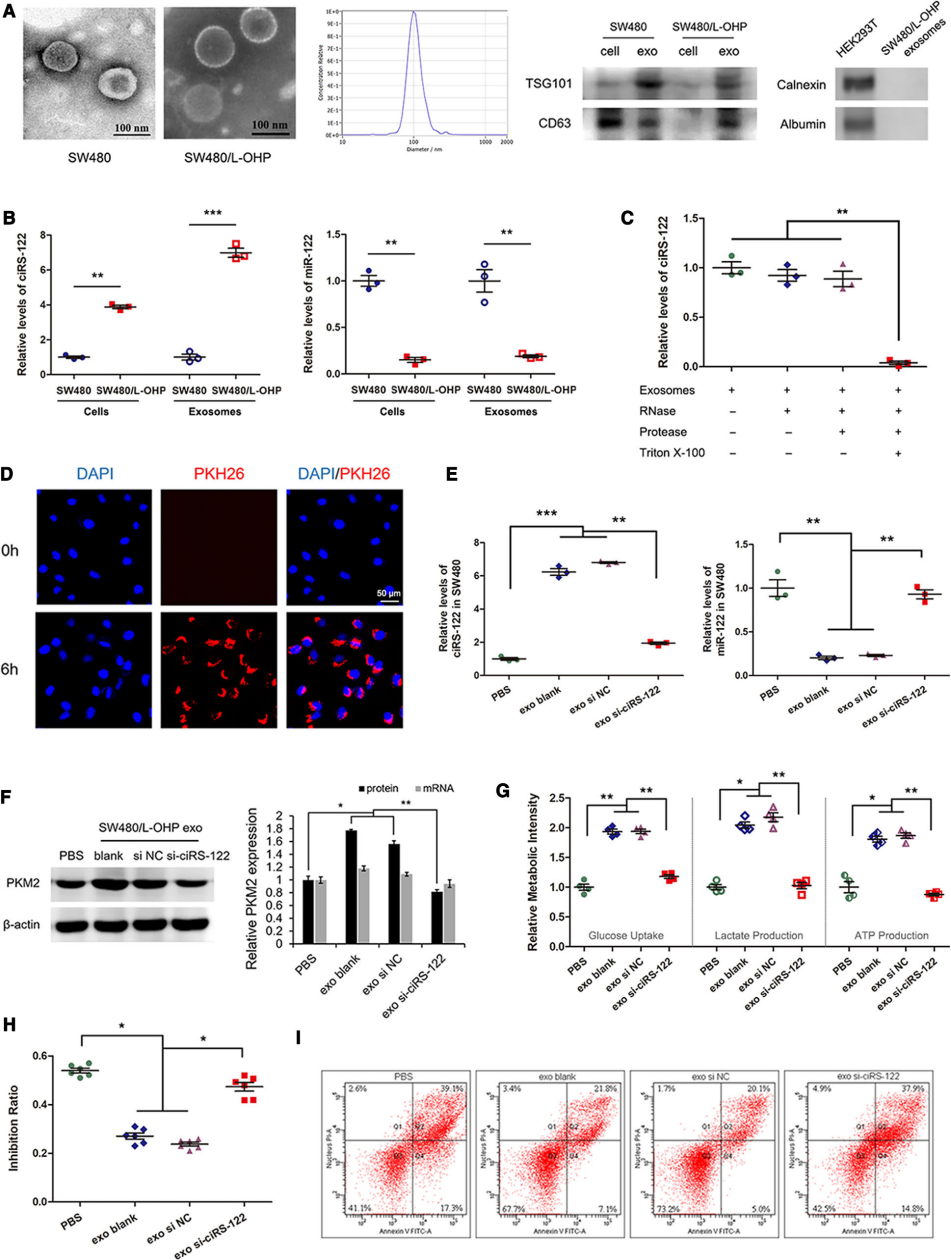
Effects of chemoresistant cell‐derived exosomes on sensitive cells. (A) TEM, NTA and WB validation of exosomes from different cells. (B) RT‐qPCR quantification of the expression levels of ciRS‐122 and miR‐122 in exosomes and cell lines (*n* = 3, mean ± SEM, *t*‐test). (C) Proteinase and RNase protection assays demonstrated that ciRS‐122 selectively resided in the exosome lumen and protected against RNase (*n* = 3, mean ± SEM, *t*‐test). (D) PKH26‐labeled SW480/L‐OHP exosomes could fuse into SW480 cells. (E) RT‐qPCR quantification of the expression levels of ciRS‐122 and miR‐122 in SW480 cells treated with diverse SW480/L‐OHP exosomes (*n* = 3, mean ± SEM, *t*‐test). (F) The expression of PKM2 protein by WB and mRNA by RT‐qPCR in SW480 cells treated with diverse SW480/L‐OHP exosomes (*n* = 3, mean ± SEM, *t*‐test). (G) Relative glucose uptake, lactate and ATP production in SW480 cells treated with diverse SW480/L‐OHP exosomes (*n* = 4, mean ± SEM, *t*‐test). (H) CCK‐8 detection of the inhibition ratio by oxaliplatin in SW480 cells treated with diverse SW480/L‐OHP exosomes (*n* = 6, mean ± SEM, *t*‐test). (I) Flow cytometry analysis of cell apoptosis by oxaliplatin in SW480 cells treated with diverse SW480/L‐OHP exosomes. *P < 0.05; **P < 0.01; ***P < 0.001.

Chemoresistance is one of the leading causes of poor prognosis in CRC. It remains unclear how drug‐sensitive and drug‐resistant clones coexist in an individual at first, but most cells become poorly responsive to chemotherapy as the tumor progresses. It was reported that exosomes could mediate intercellular signal transduction and might facilitate the alteration from drug‐sensitive cells to drug‐resistant ones. Exosomes from SW480 and the oxaliplatin‐resistant cell line SW480/L‐OHP were extracted and determined to be within a diameter of approximately 100 nm and to express the exosome markers CD63 and TSG101 but not albumin or calnexin (Fig. 2A). RNA within exosomes were quantified, and the level of ciRS‐122 was remarkably elevated, while miR‐122 was attenuated, in the exosomes from SW480/L‐OHP, similar to the expression pattern in the corresponding cells (Fig. 2B). Proteinase and RNase protection assays demonstrated that ciRS‐122 selectively resided in the exosome lumen and protected against RNase (Fig. 2C). Subsequently, exosomes from SW480/L‐OHP were labeled with PKH26 and cocultured with SW480 cells. The stained exosomes could be endocytosed into the recipient cells after 6 h (Fig. 2D) and enhanced the level of ciRS‐122 while repressing miR‐122 in response within the sensitive cell line SW480, which was eliminated when knocking down exosomal ciRS‐122 by siRNA (Fig. 2E). The expression of PKM2 protein was also upregulated due to the cocultured exosomes from chemoresistant cells, while the mRNA remained stable (Fig. 2F). As a result of the overexpression of PKM2, glycolysis in the recipient cell line SW480 became more active with increased glucose uptake, lactate and ATP production (Fig. 2G), which induced a markedly decreased inhibition ratio (Fig. 2H) and cell apoptosis by oxaliplatin (Fig. 2I). Nevertheless, exosomes with reduced ciRS‐122 slightly affected the expression of PKM2 protein and the downstream phenotypes. These results indicated that exosomes derived from oxaliplatin‐resistant cells could transfer ciRS‐122 across cells and enhance the expression level of ciRS‐122 and PKM2 protein in sensitive cells, accelerating glycolysis and drug resistance.

### 3.3 Direct regulation of chemosensitivity via the ciRS‐122/miR‐122/PKM2 axis

**Figure 3:**
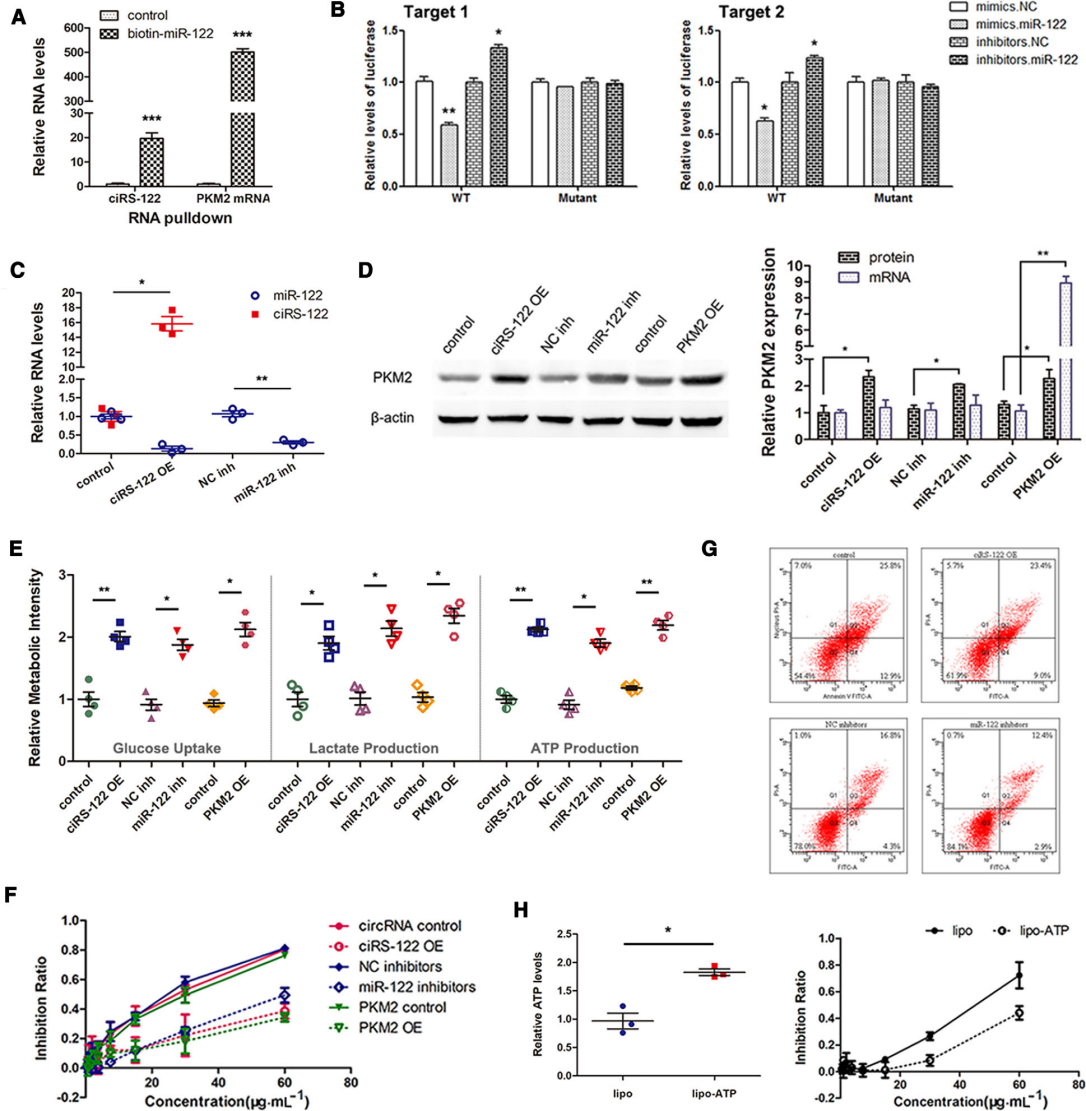
Direct regulation of chemosensitivity via ciRS‐122/miR‐122/PKM2 axis. (A) RT‐qPCR analysis of PKM2 mRNA and ciRS‐122 captured by biotin‐miR‐122 (*n* = 3, mean ± SEM, *t*‐test). (B) Direct recognition of ciRS‐122 by miR‐122 via luciferase reporter assay. For both predicted binding regions, the luciferase activity was repressed by miR‐122 mimics and increased by miR‐122 inhibitors, and the interaction was almost abolished in the mutant group (*n* = 3, mean ± SEM, *t*‐test). (C) RT‐qPCR quantification of ciRS‐122 and miR‐122 in SW480 cells treated with ciRS‐122 OE plasmid and miR‐122 inhibitors (*n* = 3, mean ± SEM, *t*‐test). (D) WB analysis of PKM2 protein and RT‐qPCR quantification of PKM2 mRNA in SW480 cells treated with ciRS‐122 or PKM2 OE plasmid and miR‐122 inhibitors (*n* = 3, mean ± SEM, *t*‐test). (E) Relative glucose uptake, lactate and ATP production in SW480 cells treated with ciRS‐122 or PKM2 OE plasmid and miR‐122 inhibitors (*n* = 4, mean ± SEM, *t*‐test). (F) CCK‐8 detection of the inhibition ratio by oxaliplatin in SW480 cells treated with ciRS‐122 or PKM2 OE plasmid and miR‐122 inhibitors. (G) Flow cytometry analysis of cell apoptosis by oxaliplatin in SW480 cells treated with ciRS‐122 OE plasmid and miR‐122 inhibitors. H. Relative ATP levels with lipo‐ATP and CCK‐8 detection of inhibition ratio by oxaliplatin in SW480 (*n* = 3, mean ± SEM, *t*‐test). *P < 0.05; **P < 0.01; ***P < 0.001.

To evaluate the possible interaction among ciRS‐122, miR‐122 and PKM2, RNA pulldown using biotin‐labeled miR‐122 was conducted first. The PKM2 mRNA and ciRS‐122 captured by biotin‐miR‐122 were found to be prominently enriched compared with the control group (CG) (Fig. 3A). A luciferase reporter assay was then performed to further demonstrate that miR‐122 could combine with ciRS‐122 via two predicted binding regions. The elevation of miR‐122 significantly repressed luciferase activity, and downregulation of miR‐122 notably increased luciferase activity, while the interaction was almost abolished in the mutant group (Fig. 3B).

Subsequently, in the drug‐sensitive SW480 cell line, ciRS‐122 or PKM2 overexpressing (OE) plasmids were utilized to enhance the expression level of ciRS‐122 or PKM2, and miR‐122 inhibitors were used to suppress miR‐122. The level of miR‐122 could also be decreased by ciRS‐122 OE plasmids (Fig. 3C). As shown in Fig. 3D, PKM2 protein was remarkably promoted by ciRS‐122 or PKM2 OE plasmids and miR‐122 inhibitors, while PKM2 mRNA changed only slightly, except for directly increasing PKM2. In accord with the upregulation of PKM2, the relative glucose uptake, lactate and ATP production were all improved (Fig. 3E). Furthermore, the reduction of miR‐122 and overexpression of ciRS‐122 or PKM2 attenuated the inhibition ratio by various doses of oxaliplatin (Fig. 3F) and cell apoptosis (Fig. 3G). To explore the actual role of ATP in the regulation of chemosensitivity, moderate ATP was transfected into SW480 by utilizing Lipofectamine 2000 to augment the intracellular ATP, which sharply reduced the drug inhibition ratio (Fig. 3H). These data further illustrated that ciRS‐122 could facilitate the expression of PKM2 as a sponge of miR‐122 at the posttranscriptional level, thereby accelerating glycolysis and lowering drug sensitivity in sensitive CRC cells.

### 3.4 **Effects of exosomes derived from chemoresistant tumors on chemosensitive tumors** *in vivo*

**Figure 4:**
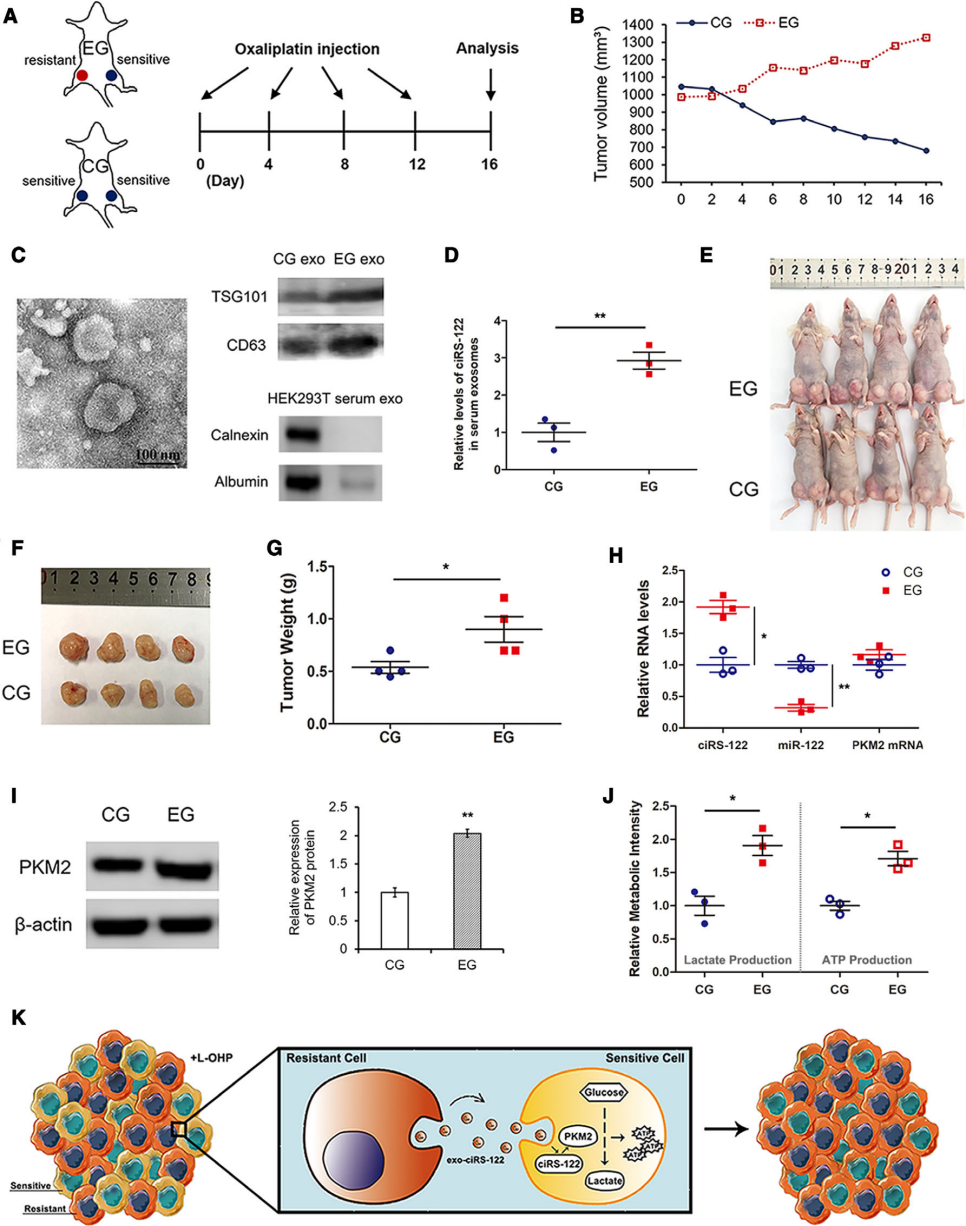
Effects of exosomes derived from resistant tumors on sensitive tumors *in vivo*. (A) A flow chart depicting the *in vivo* experimental design. (B) Alterations of tumor volume in CG and EG. (C) TEM and WB validation of exosomes from mouse serum. (D) RT‐qPCR analysis of ciRS‐122 within serum exosomes in CG and EG (*n* = 3, mean ± SEM, *t*‐test). (E) Picture of mice in both groups. (F) Picture of tumors from the left groin of mice in (E). (G) Weight of the tumors in F (*n* = 4, mean ± SEM, *t*‐test). (H) RT‐qPCR quantification of ciRS‐122, miR‐122 and PKM2 mRNA within the tumors in F (*n* = 3, mean ± SEM, *t*‐test). (I) WB analysis of PKM2 within the tumors in F (*n* = 3, mean ± SEM, *t*‐test). (J) Relative lactate and ATP levels within the tumors in F (*n* = 3, mean ± SEM, *t*‐test). (K) A proposed mechanistic model in this section. *P < 0.05; **P < 0.01.

The effects of exosome circRNA secreted from chemoresistant tumors on chemosensitive tumors *in vivo* were assessed by applying tumor‐implanted mice (*n* = 8). To achieve heterogeneity, tumor blocks from both drug‐resistant CRC cells and sensitive cells were transplanted simultaneously into every mouse in the experimental group (EG). As shown in Fig. 4A, tumor blocks from SW480/L‐OHP were implanted hypodermically in the right inguinal areas of the mice in EG, while those from SW480 cells were used in CG. On the left side, tumor blocks of equal size from SW480 were transplanted into both groups and observed closely. When tumors grew to a suitable size, 8 mg·kg^−1^ L‐OHP was intraperitoneally injected into all mice every 4 days. The volume of the left tumors was calculated via a formula in accordance with the major and minor axes recorded every 2 days. Over time, the tumors in the EG enlarged gradually, whereas those in the CG shrank (Fig. 4B). Blood from each mouse was collected followed by exosome extraction and validation (Fig. 4C). RT‐qPCR was performed to determine the high level of ciRS‐122 in serum exosomes from EG (Fig. 4D). Both groups of mice were euthanized and photographed, which indicated that the sensitive tumors on the left were notably larger in the EG than in the CG (Fig. 4E). Next, the left tumors were removed for subsequent detection. The tumors grew larger in the EG (Fig. 4F,G), which meant that chemoresistant tumors might have effects on sensitive tumors and might downregulate their drug sensitivity. As shown in Fig. 4H,I, compared with the CG, the expression levels of ciRS‐122 and PKM2 protein in tumors were remarkably increased, whereas miR‐122 was reduced, with no significant change in PKM2 mRNA. The relative lactate and ATP content were also augmented sharply in the tumors with the regulation of exosomes from drug‐resistant tumors (Fig. 4J). In summary, these findings indicate that exosomes from resistant tumors could deliver ciRS‐122 to sensitive tumors, in which glycolysis and drug resistance were enhanced by decreasing miR‐122 and upregulating PKM2 (Fig. 4K).

### 3.5 Exosome‐delivered si‐ciRS‐122 could reverse the resistance to L‐OHP *in vitro*

**Figure 5:**
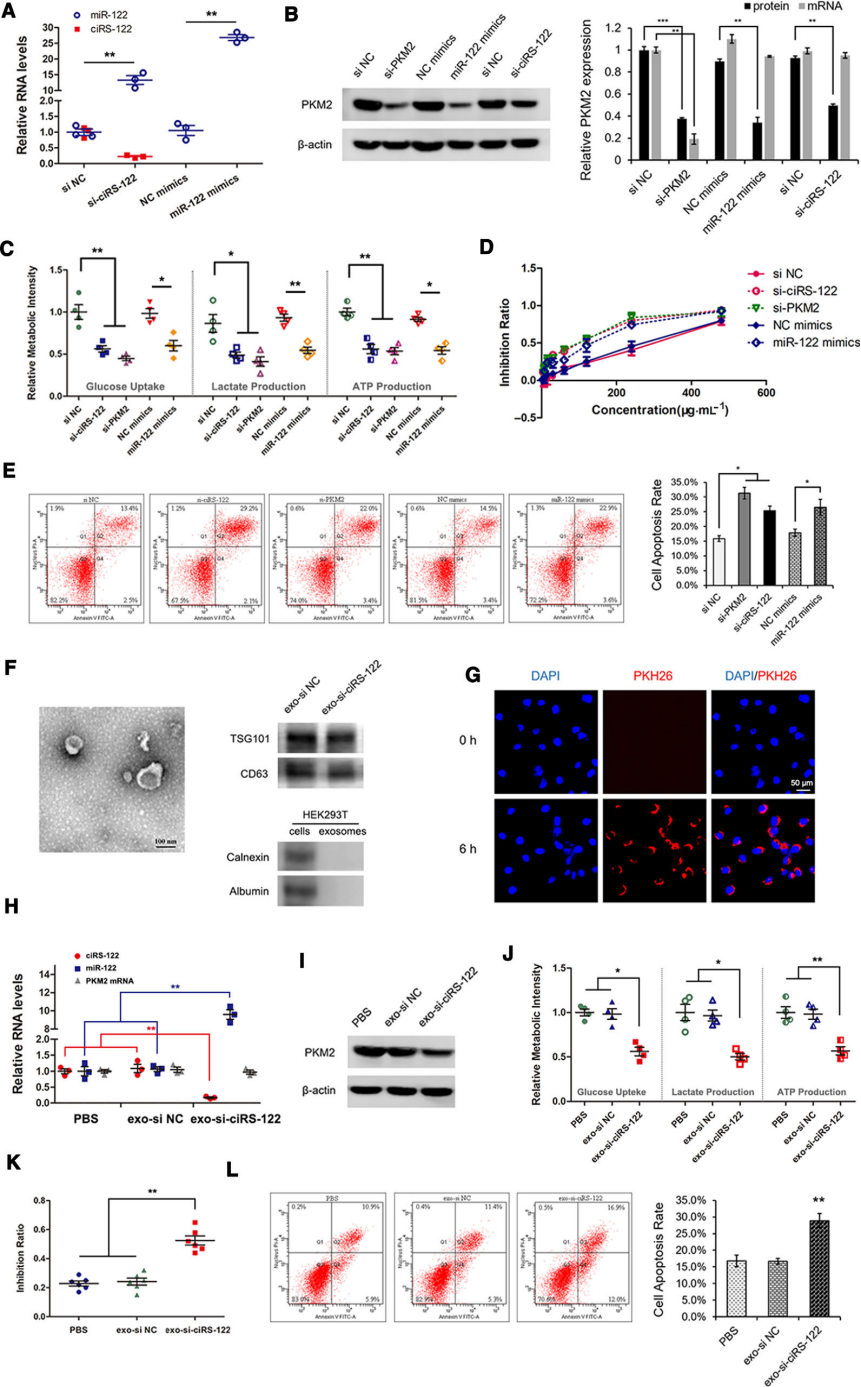
Exosome‐delivered si‐ciRS‐122 could reverse the resistance to L‐OHP *in vitro*. (A) RT‐qPCR quantification of ciRS‐122 and miR‐122 in SW480/L‐OHP cells treated with si‐ciRS‐122 or miR‐122 mimics (*n* = 3, mean ± SEM, *t*‐test). (B) WB analysis of PKM2 protein and RT‐qPCR quantification of PKM2 mRNA in SW480/L‐OHP cells treated with si‐PKM2, miR‐122 mimics or si‐ciRS‐122 (*n* = 3, mean ± SEM, *t*‐test). (C) Relative glucose uptake, lactate and ATP production in SW480/L‐OHP cells treated with si‐PKM2, miR‐122 mimics or si‐ciRS‐122 (*n* = 4, mean ± SEM, *t*‐test). (D) CCK‐8 detection of inhibition ratio by oxaliplatin in SW480/L‐OHP cells treated with si‐PKM2, miR‐122 mimics or si‐ciRS‐122. (E) Flow cytometry analysis of cell apoptosis by oxaliplatin in SW480/L‐OHP cells treated with si‐PKM2, miR‐122 mimics or si‐ciRS‐122 (*n* = 3, mean ± SEM, *t*‐test). (F) TEM and WB validation of exosomes from HEK293T cells. (G) PKH26 labeled HEK293T exosomes could fuse into SW480/L‐OHP cells. (H) RT‐qPCR quantification of ciRS‐122, miR‐122 and PKM2 mRNA with the treatment of exo‐si‐ciRS‐122 in SW480/L‐OHP cells (*n* = 3, mean ± SEM, *t*‐test). (I) WB analysis of PKM2 protein in SW480/L‐OHP cells with the treatment of exo‐si‐ciRS‐122. (J) Relative glucose uptake, lactate and ATP production with exo‐si‐ciRS‐122 in SW480/L‐OHP cells (*n* = 4, mean ± SEM, *t*‐test). (K) CCK‐8 detection of the inhibition ratio by oxaliplatin with exo‐si‐ciRS‐122 in SW480/L‐OHP cells (*n* = 6, mean ± SEM, *t*‐test). (L) Flow cytometry analysis of cell apoptosis by oxaliplatin with exo‐si‐ciRS‐122 in SW480/L‐OHP cells (*n* = 3, mean ± SEM, *t*‐test). *P < 0.05; **P < 0.01; ***P < 0.001.

Knowing the mechanism by which exosomes from drug‐resistant cells could regulate signaling pathways and mediate chemoresistance in sensitive cells, suppressing the ciRS‐122/miR‐122/PKM2 axis might be a means of reversing drug resistance in CRC. In the resistant cell line SW480/L‐OHP, siRNA of ciRS‐122 or PKM2 was transfected to downregulate the expression level of ciRS‐122 or PKM2, and miR‐122 mimics served as an enhancer of miR‐122. The level of miR‐122 could also be promoted by si‐ciRS‐122 (Fig. 5A). As exhibited** i**n Fig. 5B, PKM2 protein was remarkably reduced by si‐ciRS‐122, miR‐122 mimics and si‐PKM2, whereas PKM2 mRNA changed only slightly, except when si‐PKM2 was used. Similarly, the relative glucose uptake, lactate and ATP production were all attenuated significantly (Fig. 5C); thus, the inhibition ratio (Fig. 5D) and cell apoptosis caused by L‐OHP (Fig. 5E) were facilitated via the overexpression of miR‐122 and decrease of ciRS‐122 or PKM2. In other words, si‐ciRS‐122 could block the ciRS‐122/miR‐122/PKM2 axis at the posttranscriptional level and enhance drug sensitivity.

To further achieve the transport of si‐ciRS‐122 with better biocompatibility, si‐ciRS‐122 or its corresponding negative control (si NC) were transfected into HEK293T cells, and exosomes were isolated from the culture media (verified in Fig. 5F), delivering si‐ciRS‐122 (exo‐si‐ciRS‐122) or si NC (exo‐si NC). Exo‐si‐ciRS‐122 could fuse into SW480/L‐OHP (Fig. 5G) and abolish the expression of ciRS‐122 and PKM2 protein with elevated miR‐122 and stable PKM2 mRNA in the drug‐resistant cells (Fig. 5H,I). Glycolysis was also restrained by markedly low levels of glucose uptake, lactate and ATP production (Fig. 5J). Therefore, the drug inhibition ratio (Fig. 5K) and cell apoptosis were augmented in the resistant CRC cells (Fig. 5L). These results demonstrated that exo‐si‐ciRS‐122 could reverse the resistance to oxaliplatin by repressing the ciRS‐122/miR‐122/PKM2 pathway *in vitro*.

### 3.6 **Systemically injected exo‐si‐ciRS‐122 could sensitize the response to L‐OHP** *in vivo*

**Figure 6:**
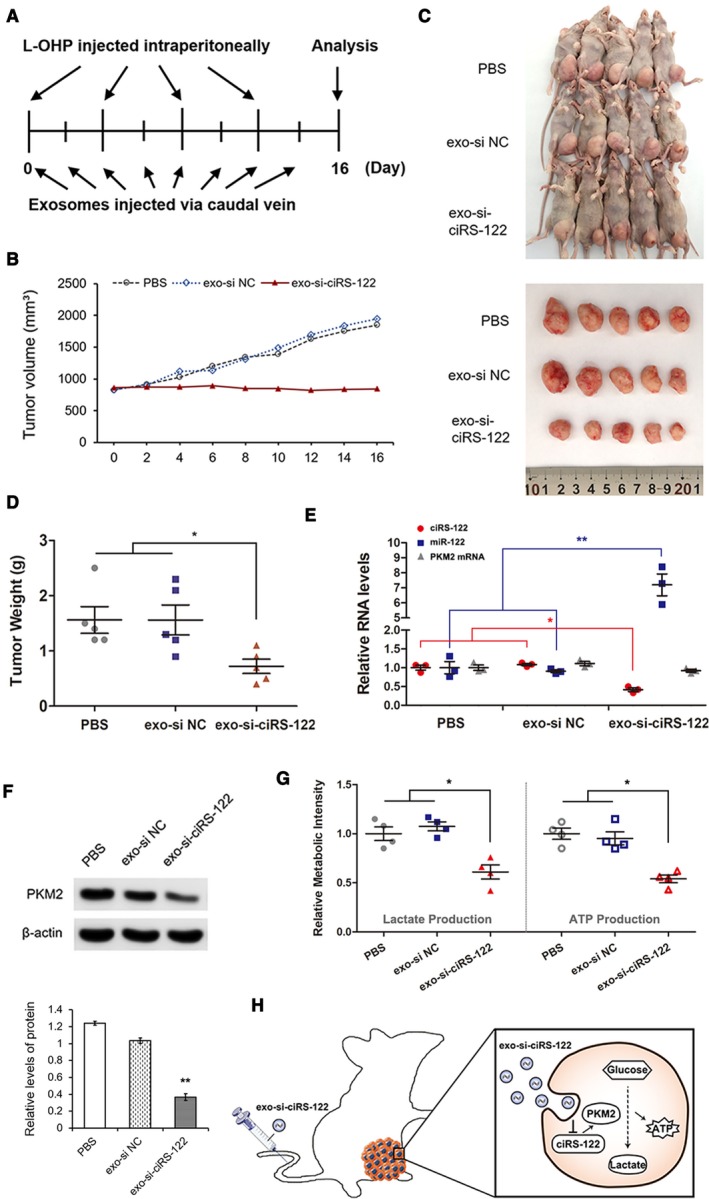
Systemically injected exo‐si‐ciRS‐122 could sensitize the response to L‐OHP *in vivo*. (A) A flow chart depicting the *in vivo* experimental design. (B) Alterations of tumor volume in different groups with or without systemically injected exo‐si‐ciRS‐122. (C) Pictures of mice and tumors in all groups. (D) Weight of the tumors in (C) (*n* = 5, mean ± SEM, *t*‐test). (E) RT‐qPCR quantification of ciRS‐122, miR‐122 and PKM2 mRNA within the tumors in (C) (*n* = 3, mean ± SEM, *t*‐test). (F) WB analysis of PKM2 within the tumors in C (*n* = 3, mean ± SEM, *t*‐test). (G) Relative lactate and ATP levels within the tumors in C (*n* = 4, mean ± SEM, *t*‐test). (H) A proposed mechanistic model in this section. *P < 0.05; **P < 0.01.

For the purpose of translational therapy, oxaliplatin‐resistant tumors were first established in nude mice (*n* = 15) with SW480/L‐OHP. Twenty micrograms of various exosomes (resuspended in 40 μL of PBS) or equivalent PBS was injected intravenously every 2 days, and 8 mg·kg^−1^ L‐OHP was injected intraperitoneally every 4 days after the tumors grew to a suitable size (Fig. 6A). The major and minor axes of tumors were measured every 2 days to calculate the volume. Over time, the tumors in the treatment group remained stable, whereas those in the two CGs became enlarged (Fig. 6B). All mice were euthanized after 2 weeks and photographed (Fig. 6C), followed by tumor removal and weighing (Fig. 6D). Tumors treated with exo‐si‐ciRS‐122 were notably smaller than those treated with exo‐si NC or PBS, which indicated a more sensitive response to L‐OHP in the treatment group. The RNA and protein of tumors were extracted and quantified to verify the low levels of ciRS‐122 and PKM2 protein in the tumors with exo‐si‐ciRS‐122, whereas the expression of miR‐122 was sharply facilitated, and PKM2 mRNA was slightly altered (Fig. 6E,F). The relative levels of lactate and ATP were also notably attenuated in the treatment group (Fig. 6G). In summary, these findings indicate that systemically injected exo‐si‐ciRS‐122 could suppress glycolysis and reverse resistance to oxaliplatin by regulating the ciRS‐122/miR‐122/PKM2 axis in CRC (Fig. 6H).

## 4 Discussion

Chemoresistance remains a considerable challenge facing treatment of CRC. Although both drug‐sensitive and drug‐resistant cells initially coexist in an individual, it remains unclear how the drug‐sensitive clones transform into the drug‐resistant ones as the tumor progresses. In our study, following confirmation of the heterogeneity in PKM2 expression and glycolysis, it was found that exosomes from oxaliplatin‐resistant cells could deliver ciRS‐122 to sensitive cells, in which glycolysis and drug resistance were enhanced by decreasing miR‐122 and upregulating PKM2. In addition, reducing ciRS‐122 could suppress glycolysis and reverse resistance to oxaliplatin *in vivo*, representing a possible alternative for the future treatment of drug‐resistant CRC.

Although research investigating means of overcoming chemoresistance in tumors is continuing, recent developments regarding ABC transporters as therapeutic targets, such as P‐gp inhibitors, RNA‐interference, nanomedicines (Bar‐Zeev *et al.*, 2017) and delivering combination strategies (Li *et al.*, 2016), have not been successful (Robey *et al.*, 2018). In this study, we focused on the amount of ATP consumed at ABC drug efflux pumps instead of blocking the transporters directly. Malignant solid tumors usually rely on aerobic glycolysis to generate ATP, including CRC (Schell *et al.*, 2014). It was previously reported that drug‐resistant cells demand an extra amount of ATP to maintain homeostasis of survival pathways under genotoxic stress compared with the corresponding drug‐sensitive cells (Zhou *et al.*, 2012). Thus, we investigated the mechanism of upstream glycolysis. When PKM2 was upregulated in the drug‐sensitive cell line SW480, glycolysis was accelerated with enhanced ATP production. In this manner, transporters might obtain more energy to pump oxaliplatin out of the cells such that the drug sensitivity is markedly weakened.

Circular RNA is an intriguing class of noncoding RNA due to its high stability and demonstrated roles in gene regulation. Several databases of circRNA were built as valuable resources for explorations of diagnostic or therapeutic targets across cancer types (Vo *et al.*, 2019; Xia *et al.*, 2018). Studies on circRNA are increasing, predominantly involving intracellular signal transduction (Han *et al.*, 2017; Zhang *et al.*, 2018) but they have largely not investigated intercellular regulation. In our study, a panel of circRNA was found to be dysregulated in the oxaliplatin‐resistant CRC cells; among these circRNA, ciRS‐122 was predicted to serve as the sponge of miR‐122. Exosomes were noted as carriers for intercellular signal transduction, delivering ciRS‐122 from drug‐resistant cells to drug‐sensitive cells, where glycolysis and drug resistance were enhanced by decreasing miR‐122 and upregulating PKM2.

Elucidating the mechanisms leading to chemoresistance is of great importance for seeking new strategies to reverse drug resistance. The application of siRNA represents a milestone in the development of precision medicine (Kedmi *et al.*, 2018). We utilized si‐ciRS‐122 to block the ciRS‐122/miR‐122/PKM2 axis and accelerated drug sensitivity. To further achieve translational therapy, exosomes from HEK293T cells were isolated to deliver si‐ciRS‐122 with better biocompatibility *in vivo*. It was demonstrated that systemically injected exo‐si‐ciRS‐122 could suppress glycolysis and reverse resistance to oxaliplatin via regulating the ciRS‐122/miR‐122/PKM2 pathway, which might help to establish a foundation for future clinical treatments.

This study has a number of limitations. The expression of PKM2 was determined to be heterogeneous even within a single tumor, but there is a lack of clinical verification of the correlation between PKM2 and chemoresistance because patients usually do not undergo surgery when they became resistant to chemotherapy in an advanced stage. Despite this limitation, this correlation was confirmed in drug‐resistant and drug‐sensitive cell lines. It was also found that the expression of upstream ciRS‐122 was positively correlated with chemoresistance at the clinical level. In addition, exosomes injected *in vivo* should be modified with some peptides to be tumor‐specific for clinical applications.

## 5 Conclusions

Exosomes from oxaliplatin‐resistant CRC cells transferred ciRS‐122 to oxaliplatin‐sensitive cells, enhancing glycolysis and drug resistance by promoting PKM2 expression. Furthermore, ciRS‐122 targeting through exosome‐delivered siRNA *in vivo* enhanced the drug response, indicating a novel potential approach for the reversion of oxaliplatin resistance in CRC.

## Conflict of interest

The authors declare no conflict of interest.

## Author contributions

XW and HZ performed all of the experiments, analyzed the data and wrote the manuscript. HY, MB, TN, TD, RL, QF, KZ, JL and YZ reviewed and edited the manuscript. YB and GY designed the experiments and reviewed the manuscript. YB is the guarantor of this work, had full access to all of the data in the study, and takes responsibility for the integrity of the data and the accuracy of the data analysis.

## Supporting information


**Fig. S1.** The heterogeneous expression of PKM2 in CRC tissues. IHC images: red arrows for high expression, blue arrows for low expression.

## References

[mol212629-bib-0001] Arnaiz E , Sole C , Manterola L , Iparraguirre L , Otaegui D and Lawrie CH (2018) CircRNAs and cancer: biomarkers and master regulators. Semin Cancer Biol 58, 90–99. 30550956 10.1016/j.semcancer.2018.12.002

[mol212629-bib-0002] Bar‐Zeev M , Livney YD and Assaraf YG (2017) Targeted nanomedicine for cancer therapeutics: towards precision medicine overcoming drug resistance. Drug Resist Updat 31, 15–30. 28867241 10.1016/j.drup.2017.05.002

[mol212629-bib-0003] Chaneton B and Gottlieb E (2012) Rocking cell metabolism: revised functions of the key glycolytic regulator PKM2 in cancer. Trends Biochem Sci 37, 309–316. 22626471 10.1016/j.tibs.2012.04.003

[mol212629-bib-0004] Han D , Li J , Wang H , Su X , Hou J , Gu Y , Qian C , Lin Y , Liu X , Huang M *et al* (2017) Circular RNA circMTO1 acts as the sponge of microRNA‐9 to suppress hepatocellular carcinoma progression. Hepatology 66, 1151–1164. 28520103 10.1002/hep.29270

[mol212629-bib-0005] Kedmi R , Veiga N , Ramishetti S , Goldsmith M , Rosenblum D , Dammes N , Hazan‐Halevy I , Nahary L , Leviatan‐Ben‐Arye S , Harlev M *et al* (2018) A modular platform for targeted RNAi therapeutics. Nat Nanotechnol 13, 214–219. 29379205 10.1038/s41565-017-0043-5

[mol212629-bib-0006] Kristensen LS , Hansen TB , Veno MT and Kjems J (2018) Circular RNAs in cancer: opportunities and challenges in the field. Oncogene 37, 555–565. 28991235 10.1038/onc.2017.361 PMC5799710

[mol212629-bib-0007] Li W , Zhang H , Assaraf YG , Zhao K , Xu X , Xie J , Yang DH and Chen ZS (2016) Overcoming ABC transporter‐mediated multidrug resistance: molecular mechanisms and novel therapeutic drug strategies. Drug Resist Updat 27, 14–29. 27449595 10.1016/j.drup.2016.05.001

[mol212629-bib-0008] Liberti MV and Locasale JW (2016) The Warburg effect: how does it benefit cancer cells? Trends Biochem Sci 41, 211–218. 26778478 10.1016/j.tibs.2015.12.001 PMC4783224

[mol212629-bib-0009] Mateescu B , Kowal EJ , van Balkom BW , Bartel S , Bhattacharyya SN , Buzás EI , Buck AH , de Candia P , Chow FW , Das S *et al* (2017) Obstacles and opportunities in the functional analysis of extracellular vesicle RNA – an ISEV position paper. J Extracell Vesicles 6, 1286095. 28326170 10.1080/20013078.2017.1286095 PMC5345583

[mol212629-bib-0010] Mathieu M , Martin‐Jaular L , Lavieu G and Thery C (2019) Specificities of secretion and uptake of exosomes and other extracellular vesicles for cell‐to‐cell communication. Nat Cell Biol 21, 9–17. 30602770 10.1038/s41556-018-0250-9

[mol212629-bib-0011] Prasetyanti PR and Medema JP (2017) Intra‐tumor heterogeneity from a cancer stem cell perspective. Mol Cancer 16, 41. 28209166 10.1186/s12943-017-0600-4 PMC5314464

[mol212629-bib-0012] Ramirez MI , Amorim MG , Gadelha C , Milic I , Welsh JA , Freitas VM , Nawaz M , Akbar N , Couch Y , Makin L *et al* (2018) Technical challenges of working with extracellular vesicles. Nanoscale 10, 881–906. 29265147 10.1039/c7nr08360b

[mol212629-bib-0013] Robey RW , Pluchino KM , Hall MD , Fojo AT , Bates SE and Gottesman MM (2018) Revisiting the role of ABC transporters in multidrug‐resistant cancer. Nat Rev Cancer 18, 452–464. 29643473 10.1038/s41568-018-0005-8 PMC6622180

[mol212629-bib-0014] Schell JC , Olson KA , Jiang L , Hawkins AJ , Van Vranken JG , Xie J , Egnatchik RA , Earl EG , DeBerardinis RJ and Rutter J (2014) A role for the mitochondrial pyruvate carrier as a repressor of the Warburg effect and colon cancer cell growth. Mol Cell 56, 400–413. 25458841 10.1016/j.molcel.2014.09.026 PMC4268416

[mol212629-bib-0015] Sharma A (2017) Chemoresistance in cancer cells: exosomes as potential regulators of therapeutic tumor heterogeneity. Nanomedicine (Lond) 12, 2137–2148. 28805111 10.2217/nnm-2017-0184

[mol212629-bib-0016] Thery C, Witwer KW , Aikawa E , Alcaraz MJ , Anderson JD , Andriantsitohaina R , Antoniou A , Arab T , Archer F , Atkin‐Smith GK *et al* (2018) Minimal information for studies of extracellular vesicles 2018 (MISEV2018): a position statement of the International Society for Extracellular Vesicles and update of the MISEV2014 guidelines. J Extracell Vesicles 7, 1535750. 30637094 10.1080/20013078.2018.1535750 PMC6322352

[mol212629-bib-0017] Verma DD , Levchenko TS , Bernstein EA and Torchilin VP (2005) ATP‐loaded liposomes effectively protect mechanical functions of the myocardium from global ischemia in an isolated rat heart model. J Control Release 108, 460–471. 16233928 10.1016/j.jconrel.2005.08.029 PMC1634739

[mol212629-bib-0018] Vo JN , Cieslik M , Zhang Y , Shukla S , Xiao L , Zhang Y , Wu YM , Dhanasekaran SM , Engelke CG , Cao X *et al* (2019) The landscape of circular RNA in cancer. Cell 176, 869–881.e813. 30735636 10.1016/j.cell.2018.12.021 PMC6601354

[mol212629-bib-0019] Wang X , Zhang H , Bai M , Ning T , Ge S , Deng T , Liu R , Zhang L , Ying G and Ba Y (2018) Exosomes serve as nanoparticles to deliver anti‐miR‐214 to reverse chemoresistance to cisplatin in gastric cancer. Mol Ther 26, 774–783. 29456019 10.1016/j.ymthe.2018.01.001 PMC5910674

[mol212629-bib-0020] Wong N , Ojo D , Yan J and Tang D (2015) PKM2 contributes to cancer metabolism. Cancer Lett 356(2 Pt A): 184–191. 24508027 10.1016/j.canlet.2014.01.031

[mol212629-bib-0021] Xia S , Feng J , Chen K , Ma Y , Gong J , Cai F , Jin Y , Gao Y , Xia L , Chang H *et al* (2018) CSCD: a database for cancer‐specific circular RNAs. Nucleic Acids Res 46, D925–D929. 29036403 10.1093/nar/gkx863 PMC5753219

[mol212629-bib-0022] Xu R , Rai A , Chen M , Suwakulsiri W , Greening DW and Simpson RJ (2018) Extracellular vesicles in cancer – implications for future improvements in cancer care. Nat Rev Clin Oncol 15, 617–638. 29795272 10.1038/s41571-018-0036-9

[mol212629-bib-0023] Zhang H , Deng T , Ge S , Liu Y , Bai M , Zhu K , Fan Q , Li J , Ning T , Tian F *et al* (2019a) Exosome circRNA secreted from adipocytes promotes the growth of hepatocellular carcinoma by targeting deubiquitination‐related USP7. Oncogene 38, 2844–2859. 30546088 10.1038/s41388-018-0619-z PMC6484761

[mol212629-bib-0024] Zhang H , Deng T , Liu R , Bai M , Zhou L , Wang X , Li S , Wang X , Yang H , Li J *et al* (2017) Exosome‐delivered EGFR regulates liver microenvironment to promote gastric cancer liver metastasis. Nat Commun 8, 15016. 28393839 10.1038/ncomms15016 PMC5394240

[mol212629-bib-0025] Zhang H , Zhu L , Bai M , Liu Y , Zhan Y , Deng T , Yang H , Sun W , Wang X , Zhu K *et al* (2019b) Exosomal circRNA derived from gastric tumor promotes white adipose browning by targeting the miR‐133/PRDM16 pathway. Int J Cancer 144, 2501–2515. 30412280 10.1002/ijc.31977

[mol212629-bib-0026] Zhang M , Zhao K , Xu X , Yang Y , Yan S , Wei P , Liu H , Xu J , Xiao F , Zhou H *et al* (2018) A peptide encoded by circular form of LINC‐PINT suppresses oncogenic transcriptional elongation in glioblastoma. Nat Commun 9, 4475. 30367041 10.1038/s41467-018-06862-2 PMC6203777

[mol212629-bib-0027] Zhou Y , Tozzi F , Chen J , Fan F , Xia L , Wang J , Gao G , Zhang A , Xia X , Brasher H *et al* (2012) Intracellular ATP levels are a pivotal determinant of chemoresistance in colon cancer cells. Cancer Res 72, 304–314. 22084398 10.1158/0008-5472.CAN-11-1674 PMC3601736

