# Supplementary figures and images for: Exosome‐delivered circRNA promotes glycolysis to induce chemoresistance through the miR‐122‐PKM2 axis in colorectal cancer
Source: Mol Oncol. 2020 Jan 24;14(3):539–55. doi: 10.1002/1878-0261.12629 (PMC7053238; doi:10.1002/1878-0261.12629)

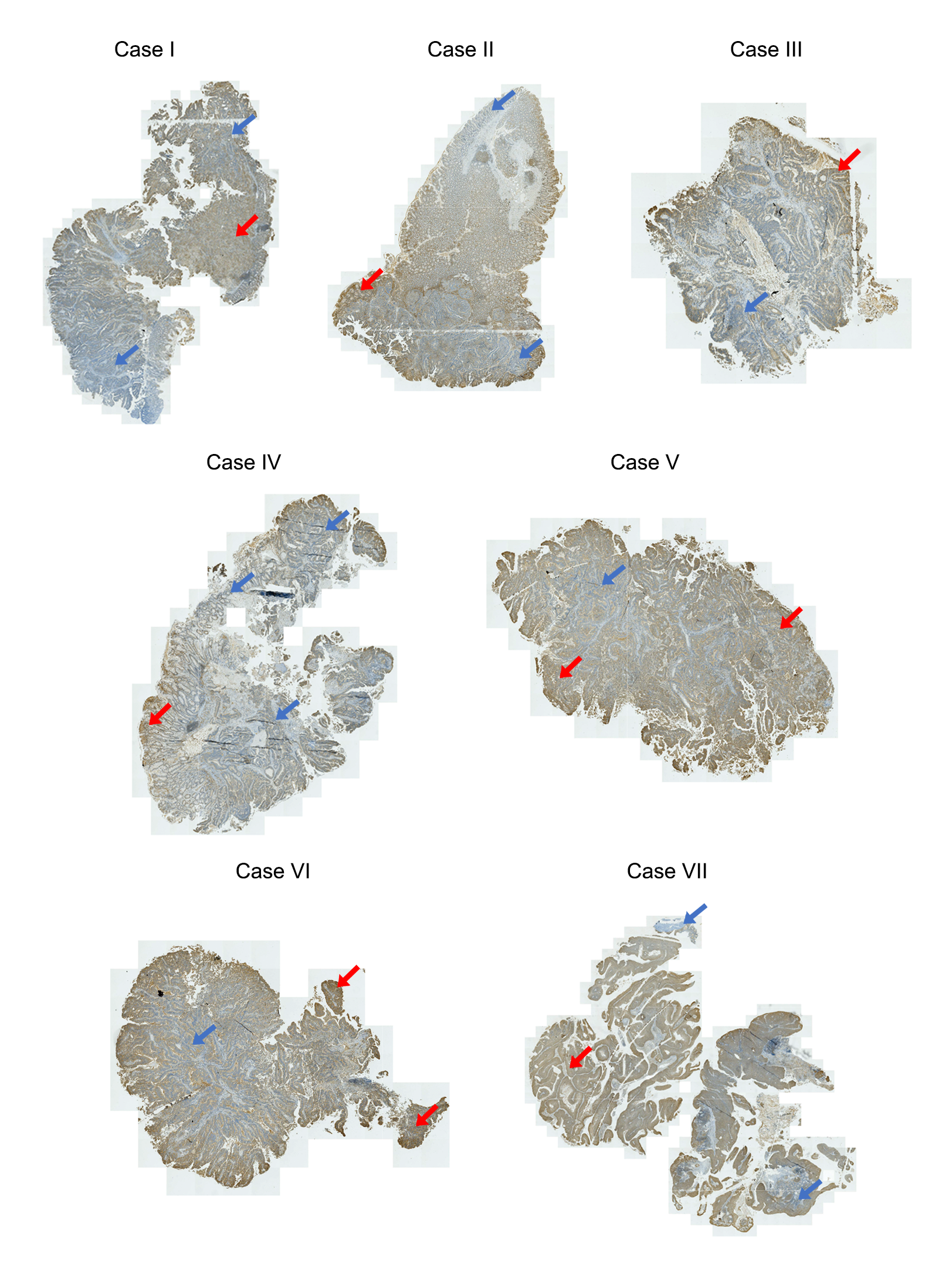

Supplement: Supplementary file 1 — Fig. S1. The heterogeneous expression of PKM2 in CRC tissues. IHC images: red arrows for high expression, blue arrows for low expression. [file MOL2-14-539-s001.tif]
